# Generation of Mouse Parthenogenetic Epiblast Stem Cells and Their Imprinting Patterns

**DOI:** 10.3390/ijms20215428

**Published:** 2019-10-31

**Authors:** Bong Jong Seo, Hyun Sik Jang, Hyuk Song, Chankyu Park, Kwonho Hong, Jeong Woong Lee, Jeong Tae Do

**Affiliations:** 1 Department of Stem Cell and Regenerative Biotechnology, Konkuk Institute of Technology, Konkuk University, 120 Neungdong-ro, Gwangjin-gu, Seoul 05029, Korea; bjseo@konkuk.ac.kr (B.J.S.); arctrus@konkuk.ac.kr (H.S.J.); songh@konkuk.ac.kr (H.S.); chankyu@konkuk.ac.kr (C.P.); hongk@konkuk.ac.kr (K.H.); 2 Research Center of Integrative Cellulomics, Korea Research Institute of Bioscience and Biotechnology, Daejeon 305-806, Korea; jwlee@kribb.re.kr

**Keywords:** embryonic stem cell (ESC), epiblast stem cell (EpiSC), parthenogenesis, imprinted gene

## Abstract

Pluripotent stem cells can be established from parthenogenetic embryos, which only possess maternal alleles with maternal-specific imprinting patterns. Previously, we and others showed that parthenogenetic embryonic stem cells (pESCs) and parthenogenetic induced pluripotent stem cells (piPSCs) progressively lose the bimaternal imprinting patterns. As ESCs and iPSCs are naïve pluripotent stem cells, parthenogenetic primed pluripotent stem cells have not yet been established, and thus, their imprinting patterns have not been studied. Here, we first established parthenogenetic epiblast stem cells (pEpiSCs) from 7.5 dpc parthenogenetic implantation embryos and compared the expression patterns and DNA methylation status of the representative imprinted genes with biparental EpiSCs. We found that there were no striking differences between pEpiSCs and biparental EpiSCs with respect to morphology, pluripotency gene expression, and differentiation potential, but there were differences in the expression and DNA methylation status of imprinted genes (*H19*, *Igf2*, *Peg1*, and *Peg3*). Moreover, pEpiSCs displayed a different DNA methylation pattern compared with that of parthenogenetic neural stem cells (pNSCs), which showed a typical bimaternal imprinting pattern. These results suggest that both naïve pluripotent stem cells and primed pluripotent stem cells have an unstable imprinting status.

## 1. Introduction

The mammalian genome is inherited biparentally from oocytes (maternal) and sperm (paternal). This biparental inheritance is crucial for the normal development and survival of the embryo. Uniparental embryos can develop until the early gastrula stage but die afterwards; for instance, parthenogenetic embryos generated by the artificial activation of unfertilized oocytes die around day 10 of gestation due to the inactivation of paternal-specific imprinted genes [1,2]. Genomic imprinting is controlled epigenetically by parental-specific DNA methylation imposed during the gametogenesis and induces gene expression bias in both chromosomal homologs [3]. Imprinted genes such as *H19* and *Igf2*, which are expressed, respectively, from only the maternal or paternal alleles, are important for the regulation of normal embryonic development [4,5,6].

Pluripotent stem cells have the potential to differentiate into all three germ layers in vitro and in vivo and have the capacity for unlimited self-renewal [7,8,9]. Pluripotency can be classified into two distinct types: naïve and primed [10]. In mice, naïve pluripotent stem cells, including embryonic stem cells (ESCs) and induced pluripotent stem cells (iPSCs), are the in vivo counterparts of the blastocyst inner cell mass [11,12], while primed pluripotent stem cells, including epiblast stem cells (EpiSCs), are in a post-implantation epiblast-like state (late gastrula stage) [13,14,15]. These two types of pluripotent stem cells are distinct in many aspects, such as differentiation potential, signaling pathways regulating their self-renewal, transcriptional and epigenetic profiles, mechanism of *Oct4* regulation, energy metabolism, and chimera formation ability [16,17,18,19].

It has been suggested that genomic imprinting is unstable in pluripotent stem cells compared to that in somatic cells [20,21,22]. ESCs cultured for the long-term experience changes in imprinting; thus, different ESC lines display substantial variation in imprinting, indicating that the stable genomic imprinting state in somatic cells could be changed after pluripotential reprogramming. Reprogramming somatic cells into pluripotent stem cells is a useful strategy for understanding epigenetic changes and the nature of pluripotency [23]. Previously, we generated parthenogenetic induced pluripotent stem cells (piPSCs) by reprogramming parthenogenetic neural stem cells (pNSCs). The piPSCs displayed typical naïve pluripotency, including the ability to form a germ line chimera [24], and exhibited different imprinting patterns from those of pNSCs, which display parthenogenetic imprinting patterns, characterized by completely unmethylated paternally imprinted genes (*H19* and *Igf2*) and completely methylated maternally imprinted genes (*Peg1* and *Peg3*); these parthenogenetic imprinting patterns became more biparental in the piPSCs. These previous studies clearly show that naïve pluripotent stem cells (mouse ESCs and iPSCs) exist in a distinct state of perturbed genomic imprinting [25] which may affect the expression of imprinted genes; however, it is unclear whether the change in imprinting pattern observed in naïve pluripotent cells also occurs in primed pluripotent stem cells, i.e., EpiSCs.

Therefore, in this study, we established parthenogenetic EpiSCs (pEpiSCs) and investigated the expression and DNA methylation patterns of the imprinted genes. Furthermore, we investigated whether the genomic imprinting in primed pluripotent EpiSCs changes in a manner similar to that seen in naïve pluripotent ESCs, as naïve and primed pluripotency display different epigenetic states.

## 2. Results

### 2.1. Derivation of Parthenogenetic EpiSCs

**Figure 1:**
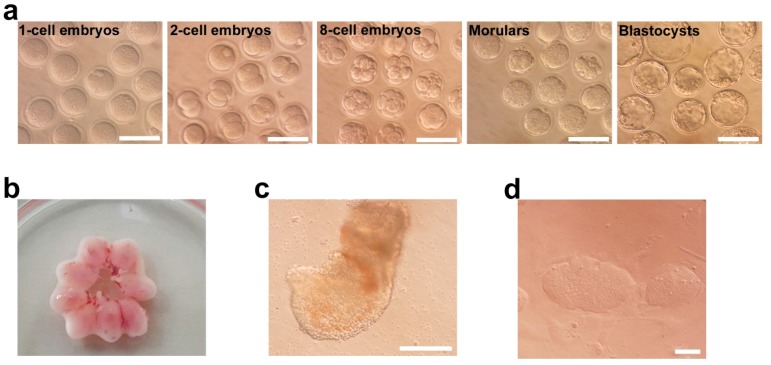
Pre- and post-implantation development of parthenogenetic embryos and generation of parthenogenetic epiblast stem cells (pEpiSCs). (**a**) Representative images of pre-implantation parthenogenetic embryos. Parthenogenetic activation resulted in two-cell embryos (79%, 27/34) and blastocysts (53%, 18/34). Scale bars represent 100 μm. (**b**) Isolated uterus containing conceptus at 7.5 dpc. (**c**) Representative image of 7.5 dpc parthenogenetic post-implantation embryo containing cup-shaped epiblasts. Scale bar represents 100 μm. (**d**) pEpiSC colonies at passage 20. Scale bar represents 50 μm.

We generated implanted parthenogenetic embryos (5.5–7.5 dpc) by artificially activating unfertilized oocytes to generate pEpiSCs. After culturing the unfertilized oocytes in CZB medium containing SrCl2 and cytochalasin B for 6 h, 79% (27/34) of the activated oocytes had developed into two-cell embryos and approximately 53% (18/34) of the parthenogenetic embryos progressed to the blastocyst stage (Figure 1a), which were transferred into pseudopregnant surrogate mice (2.5 dpc). The surrogate mice were euthanized on day 5 post-embryo transfer in order to obtain the uterus in which the parthenogenetic embryos had been implanted (Figure 1b). Parthenogenetic embryos (7.5 dpc) were successfully isolated, and from these, cup-shaped epiblasts were dissected and plated on a feeder-layered dish in EpiSC medium (Figure 1c). Epiblast outgrowths were passaged and eventually established as pEpiSCs (Figure 1d). This suggests that pEpiSCs can be established from 7.5 dpc parthenogenetic embryos and can be maintained under specific conditions.

### 2.2. pEpiSCs Maintained Primed Pluripotency and Differentiation Potential

**Figure 2:**
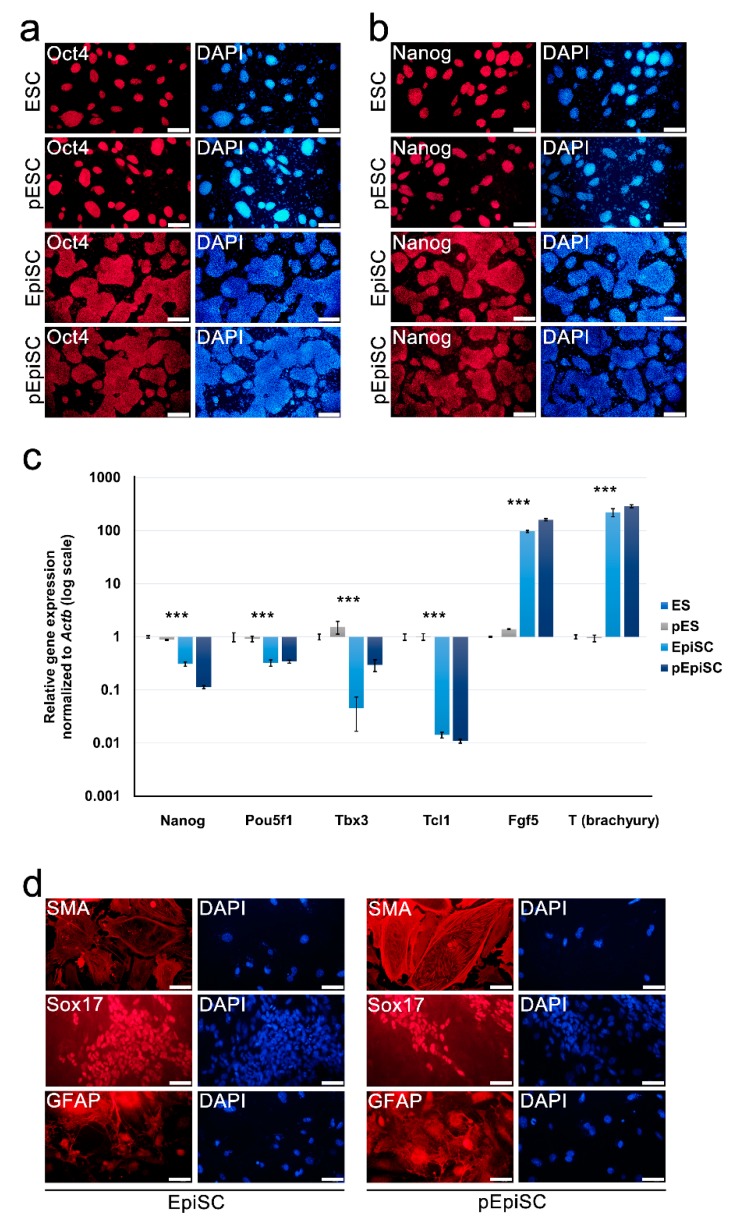
Pluripotency and differentiation potential of pEpiSCs, EpiSCs, parthenogenetic embryonic stem cells (pESCs), and ESCs. (**a,b**) Immunocytochemistry using anti-Oct4 and anti-Nanog antibodies in pEpiSCs, EpiSCs, pESCs, and ESCs. All parthenogenetic and biparental pluripotent stem cell types expressed the core pluripotency markers Oct4 (**a**) and Nanog (**b**). Nuclei were stained with DAPI (blue). Scale bars represent 200 μm. (**c**) Real-time RT-PCR analysis of pEpiSCs, EpiSCs, pESCs, and ESCs for the expression of naïve and primed pluripotency-related genes. Data are presented as the mean ± SEM for *n* = 3 independent experiments. *** *p*-value < 0.001 (**d**) In vitro differentiation by embryoid body formation with pEpiSCs and EpiSCs. Both pEpiSCs and EpiSCs could differentiate into mesoderm (smooth muscle actin, SMA), endoderm (Sox17), and ectoderm (glial fibrillary acidic protein, GFAP) lineages. Scale bars represent 100 μm.

As the established pEpiSCs formed flat colonies, typical of primed pluripotent EpiSCs (Figure 1d), we compared the expression of pluripotency genes in pEpiSCs with that of naïve (ESCs and pESCs) and normal primed (biparental EpiSCs) pluripotent stem cells. Immunocytochemistry showed that the representative pluripotency markers Oct4 and Nanog were expressed in pEpiSCs, EpiSC, pESCs, and ESCs (Figure 2a,b), although their RNA levels were relatively low in primed pluripotent pEiSCs and EpiSCs (Figure 2c). To verify the expression patterns of naïve and primed pluripotency genes, we performed real-time RT-PCR (Figure 2c). The naïve pluripotency genes *Tbx3* and *Tcl1* were expressed at lower levels in EpiSCs and pEpiSCs than in ESCs and pESCs (Figure 2c), whereas primed pluripotency genes, such as *Fgf5* and *T* (*brachyury*), were overexpressed in EpiSC and pEpiSCs compared with their levels in ESCs and pESCs (Figure 2c). Collectively, there was no clear difference between pEpiSC and EpiSCs with respect to the expression of primed pluripotency-related genes. However, there was a difference in the expression of *Tbx3*, a naïve pluripotency-related gene, which was expressed at higher levels in pEpiSCs and pESCs than in EpiSCs and ESCs; thus, *Tbx3* may be expressed at higher levels in parthenogenetic cells. However, we cannot explain why.

Next, we investigated whether pEpiSCs displayed pluripotent differentiation abilities by in vitro differentiation via embryoid body formation. It was observed that both pEpiSCs and EpiSC could differentiate into three germ layers as the differentiated cells were positive for SMA (smooth muscle actin, mesoderm), Sox17 (primitive and definitive endoderm, endoderm), and GFAP (astrocyte, ectoderm) (Figure 2d). Collectively, these results suggest that pEpiSCs display characteristics of primed pluripotent cells in terms of gene expression pattern and pluripotent differentiation in vitro.

### 2.3. Expression and Methylation Patterns of Imprinted Genes in pEpiSCs

**Figure 3:**
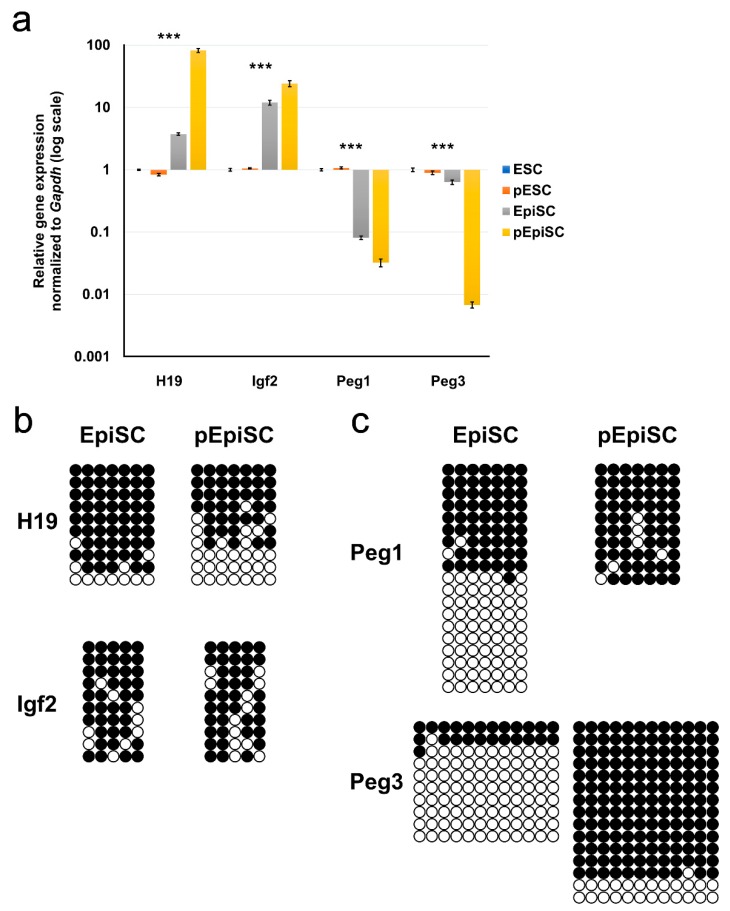
Expression and DNA methylation status of imprinted genes in parthenogenetic and biparental pluripotent stem cells. Analyses of imprinted gene expression and bisulfite genome sequencing. (**a**) Real-time RT-PCR analysis of pEpiSCs, EpiSCs, pESCs, and ESCs for paternally (*H19* and *Igf2*) and maternally (*Peg1* and *Peg3*) imprinted genes. ESCs and pESCs expressed similar levels of all four imprinted genes. *H19* and *Igf2* were expressed at higher levels in pEpiSCs than in EpiSCs (~22.11- and 2.03-fold, respectively), whereas *Peg1* and *Peg3*, were expressed at higher levels in EpiSCs than in pEpiSCs (~2.52- and 93.01-fold). Data are presented as the mean ± SEM for *n* = 3 independent experiments. *** *p*-value < 0.001. (**b**) Bisulfite DNA sequencing analysis of paternally imprinted genes (*H19* and *Igf2*) in pEpiSCs and EpiSCs. The DNA methylation level of *H19* in pEpiSCs (~57.14%) was much lower than that in EpiSCs (~84.28%), whereas similar DNA methylation levels were observed in *Igf2* (76% vs. 82%, respectively). Black and white circles represent methylated and unmethylated CpGs, respectively. (**c**) Bisulfite DNA sequencing analysis of paternally imprinted genes (*Peg1* and *Peg3*) in pEpiSCs and EpiSCs. *Peg1* and *Peg3* differentially methylated regions (DMRs) were almost completely methylated in pEpiSCs; however, the EpiSCs displayed a differentially methylated pattern (composed of completely methylated and completely unmethylated alleles) typical of imprinted genes in somatic cells. Black and white circles represent methylated and unmethylated CpGs, respectively.

Imprinted genes are expressed from either the paternal or maternal allele via the control of parent-of-origin epigenetic marks [26] and have crucial roles in embryonic development, as parthenogenic (bimaternal) and androgenic (bipaternal) mouse embryos die around the blastocyst/implantation stage [1]. Therefore, we investigated the expression of well-known paternally (*H19* and *Igf2*) and maternally (*Peg1* and *Peg3*) imprinted genes using real-time RT-PCR in EpiSCs and pEpiSCs and compared their expression to that in ESCs and pESCs. In mice, *Igf2* and *H19* are adjacent and have a common differentially methylated region (DMR) whose DNA methylation patterns regulate their expression. Although both *Igf2* and *H19* are paternally methylated, *Igf2* is only actively expressed from the paternal allele, whereas *H19* is only actively expressed from the maternal allele; thus, *Igf2* is a paternally-imprinted paternally-expressed gene and *H19* is a paternally-imprinted maternally-expressed gene. Conversely, *Peg1* and *Peg3* are maternally-imprinted paternally-expressed genes. First, we investigated the expression of the paternally (*H19* and *Igf2*) and maternally (*Peg1* and *Peg3*) imprinted genes in pEpiSCs, EpiSCs, ESCs, and pESCs (Figure 3a). Interestingly, ESCs and pESCs expressed similar levels of all four imprinted genes (Figure 3a), suggesting that the expression of imprinted genes may converge on a certain level in the naïve pluripotent state, regardless of a biparental or uniparental (bimaternal) inheritance. However, there was a clear difference between pEpiSCs and EpiSCs with respect to the expression levels of imprinted genes (Figure 3a). Paternally imprinted genes *H19* and *Igf2* were expressed at higher levels in pEpiSCs than in EpiSCs (22.11- and 2.03-fold, respectively), whereas maternally imprinted genes *Peg1* and *Peg3* were expressed at higher levels in EpiSCs than in pEpiSCs (2.52- and 93.01-fold, respectively).

We also investigated the DNA methylation status of DMRs in the imprinted genes of EpiSCs and pEpiSCs (Figure 3b,c). As reported previously [27], the DMR methylation status of *Igf2* did not exactly correspond with its expression levels (Figure 3a,b); however, the DNA methylation levels of *H19* were considerably lower in the pEpiSCs (~42.85%) than in the EpiSCs (~84.28%), corresponding with a 22.11-fold difference in expression levels. Clear differences were also observed in the expression levels of *Peg1* and *Peg3*, whose DMRs were almost fully methylated in the pEpiSCs but differentially methylated in the EpiSCs (Figure 3c), which correlated with their expression levels. Collectively, these results show that there were differences between the expression and DNA methylation patterns of imprinted genes in biparental EpiSCs and bimaternal pEpiSCs.

Next, we compared the DNA methylation status of pEpiSCs and pNSCs to verify whether a change in imprinting pattern also occurs in primed pluripotent stem cells. It has been suggested that genomic imprinting is unstable in pluripotent stem cells and changes during in vitro culture, whereas genomic imprinting in somatic cells remains stable [20]. In pNSCs, the DMRs of *H19* and *Igf2* were completely unmethylated, whereas those of *Peg1* and *Peg3* were completely methylated, which is the typical pattern observed in parthenogenetic imprinted genes (Figure S1). pEpiSCs displayed distinctly different DNA methylation patterns in the DMRs of *H19*, *Igf2*, *Peg1*, and *Peg3*, with the DMRs of *H19* and *Igf2*—which were completely unmethylated in pNSCs—showing hypermethylation (57 and 76%, respectively) and the DMRs of *Peg1* and *Peg3* showing a lesser change in the DNA methylation levels (Figure S1). The pEpiSC imprinting patterns should not have changed from the typical parthenogenetic patterns of pNSCs if the primed pluripotent state did not change the imprinted genes. Thus, these results suggest that both naïve and primed pluripotent stem cells have an unstable imprinting status and display a tendency to lose typical DNA methylation patterns in imprinted genes.

## 3. Discussion

In this study, we first established pEpiSCs and characterized the expression and DNA methylation patterns of imprinted genes present in these cells. Parthenogenetic embryos are known to be lethal at around 10.5 dpc due to unbalanced expression of imprinted genes [2,26,28]. We successfully established pEpiSCs using 7.5 dpc parthenogenetic embryos before the embryonic lethal stage. Instead of the conventional medium [14], we used a chemically-defined medium [29] that allowed us to establish and maintain the homogeneity of pEpiSC colonies.

Initially, we hypothesized that there would be some abnormal phenotypes, such as poor proliferation rate and differentiation inefficiency, due to the unbalanced expression of imprinted genes; however, we could not observe any clear differences between pEpiSCs and biparental EpiSCs with respect to the proliferation rate and the efficiency of differentiation. The pluripotency genes, *Oct4* and *Nanog*, and the primed pluripotency-specific genes, *Fgf5* and *T*, were expressed at similar levels in the pEpiSCs and control EpiSCs for over 20 passages. In addition, there was no difference between the two with respect to their embryoid body formation efficiency and differentiation into three germ layers. These results suggest that the mono-allelic expression of imprinted genes may not affect the proliferation or differentiation abilities of pEpiSCs in vitro. Though no difference was observed between pEpiSCs and EpiSCs with respect to the expression of pluripotency markers and their differentiation potential, differences were observed with respect to the expression and DNA methylation patterns of imprinted genes; these differences were not observed between pESCs and ESCs. Previously, we showed that the naïve pluripotent state resets parthenogenetic imprinting, thereby causing pESCs and ESCs to display similar DNA methylation patterns [25,30]. Although we cannot provide a clear explanation for this phenomenon, the naïve pluripotent state may reset DNA methylation and imprinting patterns more strongly than the primed pluripotent state.

By comparing pNSCs and pEpiSCs, we found that primed pluripotent stem cells could also change the DNA methylation status of imprinted genes. Initially, we hypothesized that genomic imprinting changes would be limited to the naïve pluripotent stem cells, as naïve and primed pluripotent stem cells display distinct epigenetic states. For instance, female naïve pluripotent stem cells do not contain an inactive X chromosome (Xi) and thus have two active X chromosomes (XaXa), whereas female primed pluripotent stem cells contain Xi (XaXi) in a manner similar to that observed in differentiated somatic cells [31]. The imprinted gene *Xist*, which is expressed in female somatic cells and is responsible for X chromosome inactivation, is downregulated after reprogramming to the naïve pluripotent state, i.e., iPSCs [32,33,34]. Several groups have also suggested that loss of imprinting is specific to naïve pluripotent stem cells and is rarely seen in primed pluripotent stem cells [35,36,37]. Intriguingly, our results showed that both naïve and primed pluripotent stem cells have the tendency to lose the imprinted state.

Furthermore, *H19* and *Igf2* were more highly expressed in primed pluripotent stem cells (EpiSCs and pEpiSCs) than in naïve pluripotent stem cells (ESCs and pESCs), whereas *Peg1* and *Peg3* were expressed at low levels in primed pluripotent stem cells. These results indicate that naïve and primed pluripotent stem cells may display distinct imprinted gene expression patterns that act as a distinguishing feature between the two pluripotent stem cell types. Further studies on global imprinting patterns are needed to better understand parthenogenetic primed pluripotent stem cells.

## 4. Materials and Methods

### 4.1. Generation of Parthenogenetic Epiblast Stem Cells

Super-ovulation was induced in B6D2F1 mice by serially injecting 10 IU of pregnant mare serum gonadotropin and 12 IU of human chorionic gonadotropin (hCG) 48 h later. After 14 h, the cumulus-oocyte mass was obtained from the oviduct, and the cumulus cells in the mass were digested by 0.1% hyaluronidase prepared in mouse embryonic fibroblast (MEF) medium supplemented with 15% fetal bovine serum (FBS). Separated oocytes were stabilized in CZB medium for 1 h and then incubated for 6 h in CZB medium supplemented with 10 mmol/L of strontium chloride and 5 μg/mL of cytochalasin B for parthenogenetic activation. The activated oocytes were cultured in G1 medium for 2 days at 37 °C and 5% CO_2_, and then in G2 medium. Developing blastocysts from the parthenogenetically activated embryos were transferred into the oviducts of pseudopregnant mice. After 3–4 days, developing epiblasts were isolated from the transferred embryos and attached to mitomycin C-treated MEF cells in region-selective EpiSC (rsEpiSC) medium (N2B27 supplemented with 20 ng/mL of basic fibroblast growth factor (bFGF), 2.5 μM of IWR-1, and 0.1% bovine serum albumin (BSA)) [29]. Expanded epiblast stem cells were maintained under the same conditions.

### 4.2. Cell Culture

ESCs and parthenogenetic ESCs (pESCs) were maintained in a dish layered with MEFs in mouse Embryonic Stem Cell (mESC) medium consisting of low glucose Dulbecco’s modified Eagle’s medium (D-MEM; Hyclone, 11885-084, GE Healthcare, Melbourne, VIC, Australia) supplemented with 15% heat-inactivated Fetal Bovine Serum (FBS); Hyclone), 1× penicillin/ streptomycin/glutamine (Gibco, 10378-016, Grand Island, NY, USA), 0.1 mM nonessential amino acids (Gibco, 11140-050), 1 mM β-mercaptoethanol (Gibco, 21985-023), and 103 U/mL leukemia inhibitory factor (ESGRO, Merck Millipore, Burlington, MA, USA). EpiSCs and pEpiSCs were maintained in a dish layered with MEFs in rsEpiSC medium [29].

### 4.3. Immunocytochemistry

Cells were fixed using 4% paraformaldehyde for 20 min at room temperature, washed with PBS and treated with PBS containing 0.03% Triton X-100 (Sigma, Saint Louis, MO, USA) for 5 min, and then blocked for 1 h with PBS containing 3% BSA at room temperature. The cells were then probed with primary antibodies against Pou5f1 (Oct4; monoclonal, 1:200, Abcam sc-9081, Cambridge, UK), Nanog (monoclonal, 1:200, Abcam ab80892), glial fibrillary acidic protein (GFAP; polyclonal, 1:1000, Abcam ab7260), smooth muscle actin (SMA; monoclonal, 1:200, Abcam ab7817), and Sox17 (polyclonal, 1:200, R&D Systems AF1924, Minneapolis, MN, USA). Finally, the cells were labeled with secondary antibodies conjugated to Alexa Fluor 488 or 568 (Molecular Probes, Eugene, OR, USA) in accordance with the manufacturer’s instructions.

### 4.4. RNA Isolation and Real-Time PCR Analysis

Total RNA was isolated using an RNeasy Mini Kit (Qiagen, Venlo, Netherlands) and treated with DNase to remove genomic DNA contaminants according to the manufacturer’s instructions. cDNA was synthesized from 1 mg of total RNA using a SuperiorScript III cDNA Synthesis Kit (Enzynomics, Daejeon, Korea). For real-time PCR, standard curves were created for each target gene primer set using known quantities of total cDNA from other cells. PCR reactions were performed in triplicate using TOPreal^TM^ qPCR 2x PreMIX (Enzynomics) on a Roche LightCycler 480. Target genes were amplified using 45 cycles of 95, 60, and 72 °C for 10 s each, with real-time PCR primers for *H19* (sense, 5′-CGA TTG CAC TGG TTT GGA -3′ and antisense, 5′-CTC AGA CGG AGA TGG ACG A-3′), *Igf2* (sense, 5′-GGA TCC CAG AAC CCA AGA AGA-3′ and antisense, 5′-GGG CGG CTA TTG TTG TTC TCA-3′), *Peg1* (sense, 5′-CCG CGG TCC ACA GTG TCG ATT C-3′ and antisense, 5′-GGG GGA GGT AAT ACA GGG AGG CTA-3′), *Peg3* (sense, 5′-TAC GAA TGC AAA GAT TGC GGC CAG-3′ and antisense, 5′-TGG GCA GTG GCA GCT ACT ATT TCT-3′), and *ACTB* (sense, 5′-CGC CAT GGA TGA CGA TAT CG-3′ and antisense, 5′-CGA AGC CGG CTT TGC ACA T G-3′). *ACTB* was used as a reference. We corrected for differences in PCR efficiency between the target and reference loci by making efficiency correction using the Relative Quantification Software (Roche LC 480, Roche, Basel, Switzerland).

### 4.5. Bisulfite Genome Sequencing

Genomic DNA was treated with an EZ DNA Methylation^TM^ kit (Zymo research, Irvine, USA) according to the manufacturer’s instructions and amplified by a two-step nested PCR using bisulfate PCR primers for *H19* (1st round: sense, 5′-TAA GGA GAT TAT GTT TAT TTT TGG A-3′ and antisense, 5′-CCC CCT AAT AAC ATT TAT AAC CCC-3′; 2nd round: sense, 5′-AAG GAG ATT ATG TTT ATT TTT GGA-3′ and antisense, 5′-AAA CTT AAA TAA CCC ACA ACA TTA CC-3′), *Igf2* (1st round: 5′-TTT AAT ATG ATA TTT GGA GAT AGT T-3′ and antisense, 5′-AAA AAA CAA CCT AAT ATA AAA AAA C-3′; 2nd round: sense, 5′-GAG TTT AAA GAG TTT AGA GAG GTT AAA -3′, and antisense, 5′-TAA ACT ATC CCT ACT CAA AAA AAA-3′), *Peg1* (1st round: sense, 5′-TAG GGG TTT GTT TGT TGT TTA TTT-3′ and antisense, 5′-AAC CTA TAA ATA TCT TCC CAT ATT C-3′; 2nd round: sense, 5′-GAT ATG ATA GAA AAT ATT TTG AAA TTA AAA-3′ and antisense, 5′-TAA AAA TAC CAA CAC CTA AAA AAA A-3′), and *Peg3* (1st round: sense, 5′-TTT TGT AGA GGA TTT TGA TAA GGA G-3′ and antisense, 5′-CAT ACT ACA AAC AAC CAA ATA ACC-3′; 2nd round: sense, 5′-TGT AGA GGA TTT TGA TAA GGA GGT G-3′ and antisense, 5′ -CAA TCT AAT ACA CCC ACA CTA AAC C-3′). Reactions consisted of initial denaturation at 95 °C for 10 min, amplification for 25 cycles (first round) and 35 cycles (second round) at 95 °C for 30 s, 55 °C for 30 s, and 72 °C for 30 s, and final extension at 72 °C for 10 min. Amplified PCR products were verified by electrophoresis on a 1% agarose gel, following which they were subcloned using a TOPcloner^TM^ TA core kit (Enzynomics, Daejeon, Korea) and sequenced with an M13 (−20) forward primer.

### 4.6. Statistical Analysis

All experiments were performed in triplicate, and data represent the mean ± standard deviation of the mean (SD). The significance of differences was assessed using one-way analysis of variance (ANOVA) with Fisher’s least significant different (LSD) post hoc for multiple comparisons. *p*-values of <0.05 were considered statistically significant.

### 4.7. Ethical Statement for Animal Use

Experiments were carried out in accordance with the approved guidelines (The Collaborative Institutional Training Initiative (CITI) Program, Animal Welfare & Ethics Course, K-2019-32691125 (6 August, 2019)), and all experimental protocols were approved by the Institutional Animal Care and Use Committee of Konkuk University. All mouse strains were bred and housed at the mouse facility of Konkuk University or bought from Orient-Bio Inc. (Gyeonggi-do, Korea). Animal welfare was overseen by the local committees. Mice were housed in a temperature-controlled room with an automated dark-light cycle and fed regularly ad libitum. Prior to oocyte harvesting, mice were euthanized using CO_2_.

## Supplementary Materials

The following are available online at https://www.mdpi.com/1422-0067/20/21/5428/s1, Figure S1: The DNA methylation profile of imprinted genes in pEpiSCs and pNSCs.
