# Supplementary material for: Generation of Mouse Parthenogenetic Epiblast Stem Cells and Their Imprinting Patterns
Source: Int J Mol Sci. 2019 Oct 31;20(21):5428. doi: 10.3390/ijms20215428 (PMC6862121; doi:10.3390/ijms20215428)
Supplement: Supplementary file 1 [file ijms-20-05428-s001.pdf]

Supplementary Information

Generation of mouse parthenogenetic epiblast stem cells and their imprinting patterns

Bong Jong Seo[1,‡], Hyun Sik Jang[1,‡], Hyuk Song[1], Chankyu Park[1], Kwonho Hong[1], Jeong Woong Lee[2], and Jeong Tae Do[1,*]

1 Department of Stem Cell and Regenerative Biotechnology, KU Institute of Science and Technology, Konkuk University, 120 Neungdong-ro, Gwangjin-gu, Seoul 05029, Republic of Korea

2 Biotherapeutics Translational Research Center, Korea Research Institute of Bioscience and Biotechnology, Daejeon 305-806, Republic of Korea

‡ These authors contributed equally to this work.

* Correspondence: dojt@konkuk.ac.kr; Tel.: 82-2-450-3673

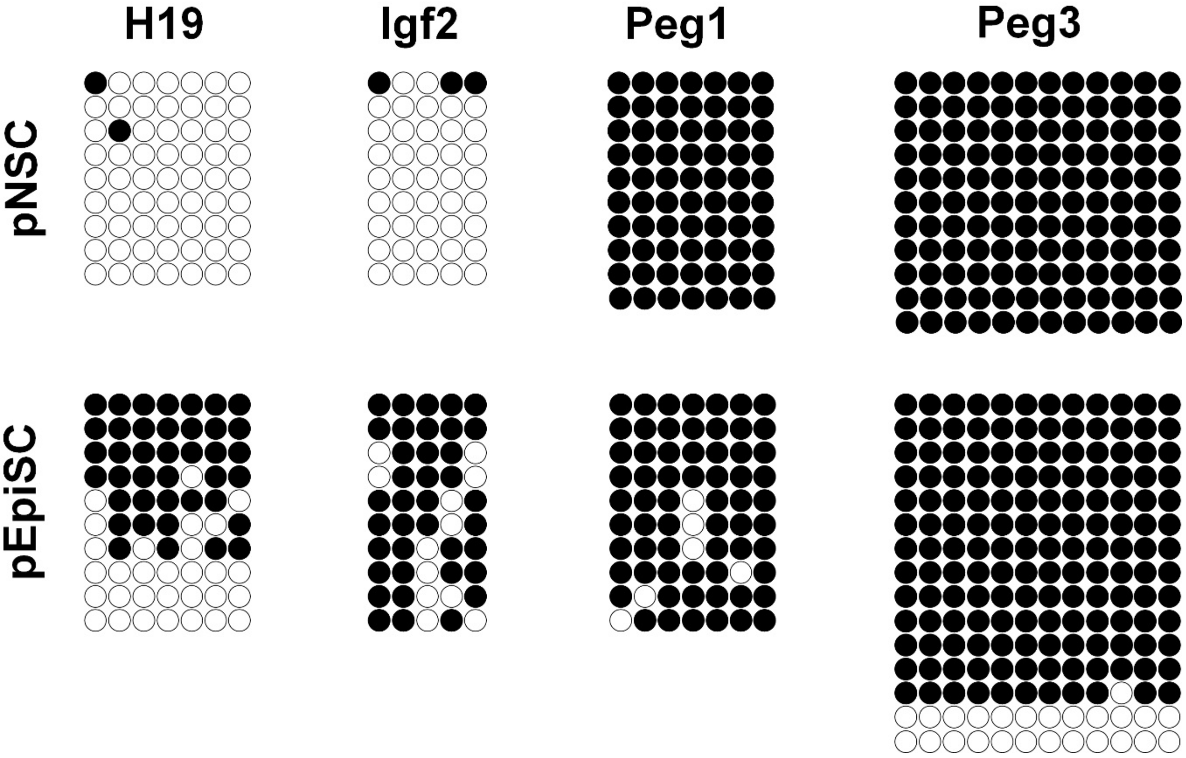

Supplementary Figure 1. The DNA methylation profile of imprinted genes in pEpiSCs and pNSCs. pNSCs displayed typical parthenogenetic DNA methylation patterns in *H19*, *Igf2*, *Peg1*, and *Peg3*; the paternally imprinted genes (*H19* and *Igf2*) were completely unmethylated, whereas maternally imprinted genes (*Peg1* and *Peg3*) were completely methylated. However, pEpiSCs showed hypermethylation patterns in *H19* and *Igf2* (57 and 76 %, respectively), and slight loss of DNA methylation in *Peg1* and *Peg3*.
